# Associations between Agility, the Relative Age Effect, Siblings, and Digit Ratio (D2:D4) in Children and Adolescents

**DOI:** 10.3390/children11080893

**Published:** 2024-07-25

**Authors:** Daniel González-Devesa, Alba López-Eguía, Lucas Amoedo, Carlos Ayán-Pérez

**Affiliations:** 1 Well-Move Research Group, Galicia Sur Health Research Institute (IIS Galicia Sur), SERGAS-UVIGO, 36310 Vigo, Spain; danidevesa4@gmail.com; 2 Facultad de Ciencias de la Educación y del Deporte, Universidad de Vigo, Campus a Xunqueira, s/n, 36005 Pontevedra, España; albaeguiax@gmail.com (A.L.-E.); lucasamoedo5a@gmail.com (L.A.); 3 Departamento de Didácticas Especiais, Universidade de Vigo, 36310 Vigo, Spain

**Keywords:** EUROFIT, finger length, physical fitness, school, students

## Abstract

Background: This study aims to analyze the influence of relative age effects, siblings, and digit ratio on the agility of children and adolescents. Methods: The study included 283 children (9.54 ± 1.36 years) and 296 adolescents (14.68 ± 1.36 years) from four different schools. The analyzed variables included anthropometric data, the presence of siblings, relative age effect, and results from the 10 × 5 m shuttle run test. Results: The findings indicated no significant association between agility and either the 2D:4D ratio or the relative age effect in both children and adolescents (*p* > 0.05). Additionally, having siblings did not have a notable impact on agility. Multiple regression analysis confirmed that relative age did not influence this lack of association (quarter of birth: *p* = 0.345, β = 0.039; siblings: *p* = 0.100, β = −0.069). However, boys showed higher performance than girls in the 10 × 5 m shuttle run test, and higher body mass index was related to lower agility. Conclusions: These findings contribute to existing knowledge on the relative effects of age and provide valuable information for physical education teachers on the influence of the 2D:4D ratio and the presence of siblings on the physical fitness of children and adolescents.

## 1. Introduction

Agility is the ability to execute rapid whole-body movements with changes in velocity or direction in response to a stimulus [1]. It is a critical component of physical fitness and is essential for many sports and activities that require rapid changes in direction, quick reflexes, and precise movements [2]. In this context, digit ratio (D2:D4), siblings, and relative age effect (RAE) have been identified as three important topics worthy of further investigation.

Digit ratio (2D:4D) refers to the relative lengths of the second and fourth digits [3]. It has been suggested that the balance of fetal testosterone and fetal estrogen influences the formation of the 2D:4D ratio, with a low 2D:4D indicating high fetal testosterone and low fetal estrogen and a high 2D:4D indicating low fetal testosterone and high fetal estrogen [4]. Several studies have tried to identify the relationship between the 2D:4D ratio and physical or sporting performance, with mixed results. For instance, Bernal et al. [5] examined the relationship between the 2D:4D ratio and VO2_max_, concluding that a low 2D:4D ratio is not associated with higher maximal oxygen uptake. In contrast, Longman et al. [6] suggest that the 2D:4D ratio can serve as a predictor of athletic fitness, linking it to athletic performance. Although there is a considerable amount of research on the 2D:4D ratio in the literature, few studies address the relationship between this ratio and agility in both children and adolescents [7,8].

Relative age refers to the subtle age differences among individuals within the same annual age group [9]. Numerous studies have confirmed the impact of the RAE on the physical fitness of children and adolescents [10,11]. Most existing research has focused on primary school children [12,13], while studies involving secondary school students are limited. Previous research suggests that primary school physical education teachers should consider the RAE when evaluating motor skills [12]. Notably, none of these studies examining the relationship between the RAE and motor competence have accounted for the presence of siblings as a confounding variable, despite their recognition as significant developmental agents [14]. 

The presence of siblings is considered a potential factor in enhancing physical fitness among children and adolescents. Older siblings provide developmentally advanced models and contribute to a stimulating, enriched environment that appears to benefit younger siblings’ development [15]. Research has shown that during childhood, having siblings is linked to higher levels of physical activity and physical fitness [16]. However, the impact of siblings on agility has been less frequently studied [17], leaving the influence of siblings on the agility of primary and secondary school students largely unexplored.

When identifying motor development, agility assessment is a key factor to be considered. Although some research has been carried out on the influence of the 2D:4D ratio on agility, no study has taken into account the presence or absence of siblings as a confounding factor. Thus, there seems to be a scientific gap that must be addressed

Therefore, this study aims to confirm whether the 2D:4D ratio and having siblings are independent factors that affect motor development, as assessed through agility both in children and adolescents.

## 2. Materials and Methods

### 2.1. Participants

This research was conducted with healthy children from four schools located in urban areas of northern Spain. The eligibility criteria required the participants to be between the ages of 7 and 18 and free from any medical conditions that might affect their ability to perform the field-based tests. Children with intellectual or physical disabilities that could interfere with their comprehension of the test procedures or proper test execution were not included in the study. Written informed consent was obtained from the parents or guardians of all participants. The study was conducted in accordance with the Declaration of Helsinki and approved by the Ethics Committee of the Faculty of Education and Sports Science at the University of Vigo.

### 2.2. Measurements

#### 2.2.1. Relative Age Effect and Siblings

The physical education teachers gathered information from the participants regarding their date of birth and the presence of siblings.

Based on birthdate, four quarterly groups were created. These groups were: Quarter 1, consisting of children born from January to March; Quarter 2, children born from April to June; Quarter 3, children born from July to September; and Quarter 4, children born from October to December. Additionally, an analysis was conducted by semesters, where participants born between January and June were analyzed as the first semester and those born between July and December as the second semester.

#### 2.2.2. Anthropometry

Body weight (kg) and body height (cm) were measured with the participants not wearing shoes and wearing only light clothing. Each child’s body mass index (BMI) was calculated using the following formula: body mass/height^2^ (kg/m^2^).

#### 2.2.3. Ratio D2:D4

Body Digit measurement expressed in centimeters (cm) was performed for digit two (2D) and digit four (4D) [18]. Digit length was directly measured from the mid-point of the proximal crease of the proximal phalanx to the distal tip of the distal phalanx for 2D and 4D on the right hand. The 2D:4D ratio was calculated by dividing 2D length by the 4D length. In addition, the right minus left 2D:4D ratio (Dr-l), suggested as an additional negative marker for prenatal testosterone, was calculated [3].

#### 2.2.4. Agility

The agility was assessed using the 10 × 5 m shuttle run test from the EUROFIT battery [19]. The 10 × 5 m shuttle run test has been shown to be reliable and valid for being administered with primary and secondary school children [20]. 

The test was conducted on a flat, non-slip surface with two parallel lines placed 5 m apart. The children were asked to stand behind one of the lines and run toward the opposite line, step on it, and then return to the starting line, completing a total of 5 rounds for each line. The time taken to complete the test was measured in seconds. 

### 2.3. Procedure

All measurements were taken between March and May 2024 during morning physical education classes. Two senior students from the Degree in Primary Education program (L.A. and A.L.-E.) and one PhD in Sport Science student (D.G.-D.), all familiar with the test protocols, performed the measurements. The physical education teachers at each school oversaw the assessment of session performance. 

During the first week, anthropometric measurements (ratio D2:D4, weight, and height) were carried out, and the protocol of the 10 × 5 m shuttle run test was explained. The 10 × 5 m shuttle run tests were performed individually during physical education sessions in the gymnasium. The children were encouraged to perform the test after their execution was observed by their teacher, and they were also allowed to perform several attempts in order to become familiar with it and avoid a learning effect. 

During the following week, the 10 × 5 m shuttle run tests were performed. The demographic data, including date of birth, gender, and presence of siblings of the participants, were provided by the physical education teachers.

### 2.4. Statistical Analysis

The statistical analysis was conducted using the Statistical Package for the Social Sciences (SPSS v24, IBM Corp., Armonk, NY, USA). After assessing normal distribution with the Kolmogorov–Smirnov test, quantitative variables were assessed as the mean (standard deviation) or median (interquartile range), and qualitative variables were assessed as *n* (%). The 10 × 5 m shuttle run scores, not following a normal distribution, were subjected to non-parametric tests for comparisons involving them. The Kruskal–Wallis test was used to compare the 10 × 5 m shuttle run test percentiles among the quarters of birth, and the Mann–Whitney U test was used to compare them between semesters. Spearman’s rank coefficient was used for correlations between variables. A correlation matrix was created to explore multicollinearity among variables, with the variance inflation factor used as a metric, applying a cut-off value of >5 [21] to identify potential multicollinearity issues. Additionally, a multiple linear regression analysis was performed to assess the interaction between the quarter of birth and the existence of siblings, with 10 × 5 m shuttle run scores as the dependent variable.

## 3. Results

A total of 579 primary school students (*n* = 283; mean age: 9.54 ± 1.36 years; 50.88% boys) and secondary school students (*n* = 296; mean age: 14.68 ± 1.36 years; 55.41% boys) completed the tests. The demographic characteristics of the participants are presented in Table 1.

No association was found between performance on the 10 × 5 m shuttle run test and chronological age for the overall sample (Rho = 0.067; *p* = 0.108), but there was an association for the girls (Rho = 0.163; *p* = 0.007). Table 2 shows the correlations between the performance in the 10 × 5 m shuttle run and the other principal variables.

Data on the RAE, categorized by educational level, are presented in Table 3. The analysis revealed that the RAE had no significant impact on 10 × 5 m shuttle run test performance. However, the results were better in the first quarter of birth (*p* > 0.005). When the data were analyzed by semester, no significant differences were found (*p* = 0.316).

Of note, 79.1% of the participants reported having at least one sibling. The comparative analysis indicated that having siblings did not significantly affect 10 × 5 m shuttle run test performance for the overall sample, as well as for both primary and secondary students, irrespective of gender. Multiple regression analysis further confirmed that the RAE did not influence this lack of association (quarter of birth: *p* = 0.345, β = 0.039; siblings: *p* = 0.100, β = −0.069).

**Figure 1:**
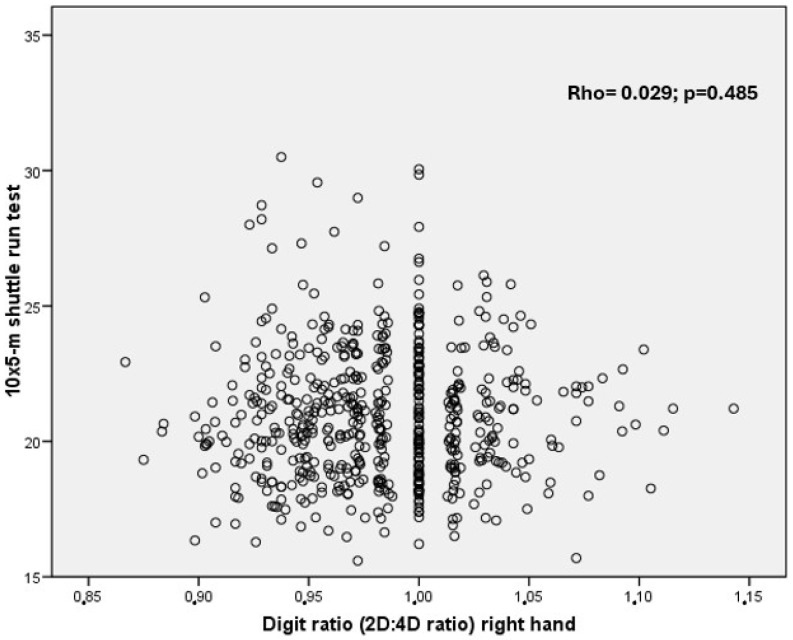
Relationship between digit ratio (2D:4D) and the 10 × 5 m shuttle run test.

Boys performed better than girls in the 10 × 5 m shuttle run test (*p* < 0.001). BMI showed a significant association with 10 × 5 m shuttle run score (Rho = 0.225; *p* < 0.001). However, there were no significant correlations between the 2D:4D ratio and the 10 × 5 m shuttle run test; see Figure 1. Multiple regression analysis confirmed that BMI and gender influence performance in the 5 × 10 m shuttle run test (Table 4).

The 5 × 10 m shuttle run regression model obtained was as follows: 5 × 10 m shuttle run score = 19.049 + 0.136 × “BMI” − 1.508 × “Gender (male = 0; female = 1)”.

## 4. Discussion

This study aimed to evaluate whether the RAE, the D2:D4 ratio, and the presence of siblings influence the agility of both primary and secondary school children. The findings from this research could offer valuable insights for physical education teachers at both primary and secondary levels, providing them with a deeper understanding of how these factors may impact children’s physical performance.

The present study did not find an association between agility and the 2D:4D ratio. This result contrasts with the findings of previous studies. Ranson et al. [7] observed that children who achieved better times in the 10 × 5 m shuttle run test had a lower 2D:4D ratio, but they acknowledged that this agility is a variable that can be modified through training. Similarly, Agha-Alinejad et al. [22] found that a lower 2D:4D ratio correlated with greater agility in pre-adolescent females. These differences could be attributed to the fact that agility is influenced by multiple factors, such as age and maturation level [23] or the activity levels [24] of the children and adolescents. Therefore, future research should consider conducting a multifactorial analysis that takes into account physical literacy and the activity levels of the younger population.

No RAE was observed in the agility levels of children and adolescents, regardless of whether the data were analyzed by quarter or by semester. Previous studies have produced mixed results regarding the impact of the RAE on various aspects of physical fitness in children. For example, Roberts et al. [25] observed that a statistically significant RAE existed in the cardiorespiratory fitness score, based on data from a large cross-sectional cohort study. Dutil et al. [13] administered the CAMSA to 8044 children aged 8–12 years and observed the presence of the RAE. Nonetheless, the significant associations identified had only negligible effect sizes. Dutil et al. [13] suggested that the RAE bias is mainly negligible with regard to the domain scores and overall Canadian assessment of physical literacy scores in a large sample of children. Regarding adolescents, our results do not indicate such a relationship and support the notion that RAEs are more pronounced in early grades [26]. Interestingly, research on the influence of relative age on fitness has shown that the RAE remains evident up to 12–14 years but tends to diminish thereafter [27] or even disappear [10]. In summary, these findings indicate no significant association between the RAE and the performance of the 10 × 5 m shuttle run test in primary and secondary school students.

Our multifactorial analysis revealed that BMI and gender may influence the agility of primary and secondary school students. These results align with previous research suggesting that boys exhibit higher levels of agility than girls in both childhood [28] and adolescence [29]. Additionally, it was observed that BMI can significantly impact agility, with a lower BMI often associated with better performance in agility tasks. Studies have consistently shown that overweight and obese adolescents tend to have lower levels of physical fitness than their non-overweight peers, irrespective of gender [29,30]. It has been suggested that intervention strategies to prevent unhealthy weight gain among children and adolescents could include enhancing locomotor skill competence as a key component [31]. Moreover, agility training has been advocated as an effective program for improving body composition in children [32]. For example, significant improvements in body composition were found after a 6-week sprint-strength and agility training program [33]. Therefore, these findings underscore the importance of considering both BMI and gender when assessing and training for agility in young populations [34]. Furthermore, integrating agility training into physical activity programs for primary and secondary school students should be strongly considered.

Various studies on infants suggest that children with older siblings exhibit higher motor skill levels [16,35], while others did not find significant associations [36,37]. Research involving older children is limited, which restricts further discussion. Notably, Lopes and Monteiro [38], reported similar findings to ours, showing no significant association between sibling characteristics and motor competence in children. Contrastingly, research indicates that siblings significantly influence physical fitness levels [39,40]. Our results also showed that the RAE does not affect the relationship between siblings and agility. This could be explained by the fact that infancy and early childhood are crucial phases for motor development, during which sibling influence and the RAE are potentially more pronounced. However, more comprehensive studies that consider the age of siblings and their level of cohabitation are needed to better understand these relationships.

### 4.1. Strengths and Limitations

The main strength of this investigation lies in its originality, as there is limited research that simultaneously examines the interplay between agility, digital ratio, and sibling presence. The findings can be particularly beneficial for physical educators who seek to identify and exclude factors that might affect the motor development of children. On the other hand, there are some methodological issues that limit the solidity of these findings. For instance, the sample size was small in comparison with other investigations assessing the RAE and fitness. Indeed, studies on the RAE have included a considerable number of participants. Cupeiro et al. [41] found a significant relationship between the RAE and fitness levels in a study conducted among 3147 preschoolers. Similarly, Roberts et al. [25] confirmed the impact of relative age on cardiorespiratory fitness after assessing more than 15,000 primary and secondary school children. It should also be acknowledged that the data were equally balanced regarding the presence or absence of siblings. In addition, the participants came from similar socioeconomic and cultural backgrounds, both of which could also play a role in motor development, thus limiting the generalizability of the findings. In this regard, Lopes and Monteiro [38] showed that socio-economic status (parents’ occupation and education levels), living space (housing type and the presence of play areas), peer interactions (age and sex of playmates, and interaction outside school), and family educational practices (play boundaries, type of toys, and time spent with each parent) significantly influence children’s motor competence. Therefore, all of these factors should be considered for a more thorough assessment in future research.

### 4.2. Practical Applications

The findings of this study offer valuable new insights for physical education teachers regarding the impact of the 2D:4D ratio, the RAE, and the presence of siblings on the physical condition of children and adolescents. These insights can guide the design of more informed and personalized educational programs. Furthermore, an association was observed between higher BMI and lower agility, suggesting that health and fitness programs may benefit from focusing on BMI control to improve youth agility. Finally, since this was a cross-sectional study, the number of participants was somewhat limited. Researchers should aim to analyze longitudinal data from epidemiological studies whenever possible to increase the sample size. Also, there have been studies where data were obtained through school fitness and physical activity surveys conducted across vast geographical areas [31] or by performing a battery of tests on children recruited through sports clubs [42] to achieve a considerable and representative sample size.

## 5. Conclusions

The results indicate that the 2D:4D ratio, the RAE, and having siblings are not significantly associated with agility levels in children and adolescents. However, boys demonstrated better performance in the 10 × 5 m shuttle run test compared to girls. Additionally, a higher BMI was associated with lower agility. This information adds to existing knowledge on the RAE and provides new valuable insights for physical education teachers regarding the impact of the 2D:4D ratio and having siblings on the physical fitness of children and adolescents.

## Figures and Tables

**Table 1:** Demographic characteristics of the participants (*n* = 579).

		*n* (%)	$\bar{X}$ ± SD	Range
Gender	Boys	308 (53.2)		
	Girls	271 (46.8)		
Age (years)		579	12.17 ± 2.91	7.22–18.05
School level	Primary school students	283 (48.9)		
	Secondary school students	296 (51.1)		
Body mass (kg)		579	47.86 ± 17.44	18.3–108.80
Height (cm)		579	152.1 ± 16.7	116–198
BMI (kg/m^2^)		579	19.99 ± 4.32	12.14–49.01
Number of siblings		579	1.19 ± 0.85	0–5
	0	121 (20.9)		
	1	265 (45.8)		
	2	161 (27.8)		
	≥3	32 (5.5)		
Digit ratio (2D:4D)		579	0.98 ± 0.04	0.87–1.14
	Length of second finger	579	6.41 ± 0.75	4.7–8.5
	Length of fourth finger	579	6.52 ± 0.74	4.8–8.5
5 × 10 m Shuttle Run Test (s)		578	20.96 ± 2.43	15.59–30.5

Abbreviations: BMI = body mass index; SD = standard deviation; $\bar{X}$ = mean.

**Table 2:** Spearman’s rank coefficients between 10 × 5 m shuttle run test scores and predictor variables.

	Rho	*p*-Value
BMI (kg/m^2^)	0.225	<0.001
Gender	−0.326	<0.001
Age (years)	0.067	0.108
Number of siblings	−0.074	0.076
Digit ratio (2D:4D)	0.029	0.485

**Table 3:** 5 × 10 m shuttle run test percentile according to quarter of birth and educational level.

	5 × 10 m Shuttle Run Test (s)					
	Primary School (*p* = 0.241)		Secondary School (*p* = 0.418)		Total (*p* = 0.515)	
Quarter of Birth	*n*	Median (IQR)	*n*	Median (IQR)	*n*	Median (IQR)
1st	77	19.99 (18.57; 22.02)	67	20.97 (19.38; 22.50)	144	20.34 (18.74; 22.27)
2nd	59	19.99 (18.26; 21.37)	76	21.55 (20.10; 22.81)	135	20.80 (19.27; 22.32)
3rd	79	20.45 (18.89; 22.34)	78	21.26 (19.83; 22.83)	157	20.83 (19.46; 22.56)
4th	67	20.73 (18.91; 22.66)	75	21.21 (19.29; 22.57)	142	20.91 (19.06; 22.59)

Abbreviations: IQR = Interquartile Range. Comparison of 5 × 10 m Shuttle Run Test percentile among quarter of birth with Kruskal-Wallis test.

**Table 4:** Multiple regression analysis predicting 5 × 10 m shuttle run test results.

Variables	R^2^	b	SE (β)	β Coefficient	F	*t*-Value	*p*-Value	VIF
Model (5 × 10 m)	0.149				50.288		<0.001	
Intercept		19.049	0.450			42.328	<0.001	
BMI (kg/m^2^)		0.136	0.022	0.242		6.281	<0.001	1.001
Gender		−1.508	0.187	−0.310		−8.054	<0.001	1.001

Abbreviations: β = standardized beta; b = non-standardized regression coefficient; SE = standard error; VIF = variance inflation factor.

## Data Availability

Data are contained within the article.
